# A Randomized Double-Blind Placebo-Controlled Clinical Study to Evaluate the Effect on the Weight of a Medical Device with Polyglucosamine L112 in a Group of Overweight and Obese Subjects

**DOI:** 10.3390/nu15163516

**Published:** 2023-08-10

**Authors:** Mariangela Rondanelli, Simone Perna, Matteo Della Porta, Federico Lombardoni, Zaira Patelli, Mara Nichetti, Clara Gasparri, Elvira Pistolesi, Benvenuto Cestaro, Roberta Cazzola

**Affiliations:** 1 Department of Public Health, Experimental and Forensic Medicine, University of Pavia, 27100 Pavia, Italy; mariangela.rondanelli@unipv.it; 2 IRCCS Mondino Foundation, 27100 Pavia, Italy; 3 Division of Human Nutrition, Department of Food, Environmental and Nutritional Sciences (DeFENS), Università degli Studi di Milano, 20133 Milano, Italy; simoneperna@hotmail.it; 4 Department of Biomedical and Clinical Sciences, University of Milano, 20157 Milano, Italy; matteodellaporta92@gmail.com (M.D.P.); federico.lombardoni@unimi.it (F.L.); roberta.cazzola@unimi.it (R.C.); 5 Endocrinology and Nutrition Unit, Azienda di Servizi alla Persona “Istituto Santa Margherita”, University of Pavia, 27100 Pavia, Italy; zaira.patelli01@universitadipavia.it (Z.P.); dietista.mara.nichetti@gmail.com (M.N.); 6 Postgraduate Course in Nutritional Food and Applied Nutrition, University of Milano, 20157 Milano, Italy; epistolesi@hunza.it (E.P.); benvenuto.cestaro@unimi.it (B.C.)

**Keywords:** polyglucosamine L112, body weight, cholesterol levels, glucosamine, insulin resistance, fat-soluble vitamins

## Abstract

Background. Overweight and obesity have reached epidemic proportions and safe treatments are needed to heal these diseases. Objective. The objective of this study is to examine the activity of a medical device based on polyglucosamine polymers (PG) on body weight (BW) reduction, insulin resistance, and the serum levels of fat-soluble vitamins and glucosamine. Methods. A double-blind placebo-controlled interventional study comparing PG and a placebo (PL) was conducted. One hundred and fifty overweight or obese cases were treated, divided into two groups for a period of 90 days at the dosage of 3 g/day. Results. One hundred and nineteen cases (58 with PG and 61 with PL, respectively) concluded the treatment. PG was more effective than the PL on the reduction of BW and insulin resistance. No modification of fat-soluble vitamins (Vit A, E, D_3_, K_1_) and glucosamine levels was shown. Total cholesterol levels were significantly more reduced in the PG group compared to the PL group as it was for subjects with a BW decrease of >5%. Conclusions. PG acts as a safe medical device, is not absorbed, and binds lipids in the upper gastrointestinal tract, reducing their availability, with a significant activity on the reduction of BW, insulin resistance, and cholesterol levels without the modification of fat-soluble vitamins.

## 1. Introduction

Obesity is a pathological condition that has currently reached epidemic proportions, with about 1.9 billion overweight adults and 650 million obese people in the world, and currently represents a growing problem of both a health and economic nature, being associated with increased mortality and morbidity, and related to the risk of diseases such as type 2 diabetes mellitus (T2DM), cardiovascular disease, and obstructive sleep apnea [1].

The treatment of obesity makes use of numerous possible means, ranging from lifestyle modification (physical exercise associated with diet therapy) to the use of drugs and to bariatric surgery, possibly in combination with each other.

Conventional therapies, mainly based on dietary intervention and the intake of drugs that act on different components of the body’s energy-balance-regulation system, while maintaining their importance, have so far led to limited results in terms of weight loss, while bariatric surgery has shown greater efficacy, especially when associated with an improvement in comorbidities (e.g., T2DM) [2].

However, surgery is a substantially valid option for extreme forms of obesity and does not seem capable of stopping the growth of the pandemic. For this reason, innovative pharmacological strategies have recently been tested that simultaneously target different factors involved in the genesis of obesity [3].

The pharmacological options available to date are few (liraglutide, orlistat, and the combination of naltrexone and bupropion) and with specific indications, such as the maintenance of weight loss in subjects with a BMI > 27 kg/m^2^ and the presence of risk factors, or in subjects with a BMI ≥ 30 kg/m^2^, preferably in association with low-calorie diets and physical exercise; furthermore, all these therapies are burdened by heavy side effects [1].

Medical devices, specifically polyglucosamine L112 (PG), are a further possibility for the treatment of overweight and obesity which have recently been proposed by the literature to be associated with nutritional intervention and physical activity.

According to the definition of the World Health Organization, a medical device is a tool intended to be used for human beings for medical purposes which does not exert its action through pharmacological, immunological, or metabolic mechanisms [4]. This is the main characteristic of PG, that can be administered daily by the oral route, and represents the only medical device among the biopolymers used to reduce body weight (BW).

PG is a chitosan positively charged in an acidic medium and which acts by forming high-affinity bonds with lipid molecules present in the gastrointestinal lumen, decreasing their bioavailability. Structurally, PG is a chitin derivative extracted from the exoskeleton of crustaceans and consisting of unbranched polymers of beta (1,4)-D-glucosamine, in which the different chains are stabilized by hydrogen bonds. The polymers contained in PG are formed by >85% of deacetylated chitin and can be considered a chitosan with a defined mean molecular weight. Furthermore, the addition of ascorbic acid and tartaric acid allows the formation of a network of polymers which are biocompatible and capable of binding fats [5,6]. In this way, large, poorly digestible lipid–chitosan emulsions are created, which are partly eliminated and partly used as a substrate by the bacteria present in the colon.

Pharmacological studies have shown that PG reduces BW and increases the fat excretion in feces [7,8]. A recent meta-analysis, including 399 subjects (ages ranging between 21 and 75 years, and a BMI between 26 and 45 kg/m^2^) followed for a period ranging from 12 weeks to 1 year, showed that PG supplementation improved weight loss by −1.78 kg [−2.78, −0.79], BMI by −1.52 kg/m^2^ [−3.58, 0.54], and improved waist-circumference reduction by −1.45 cm [−2.77, −0.12] [9].

Given this background, the aim of this randomized, double-blind, placebo-controlled study was to confirm the efficacy of PG on BW as a primary endpoint, while insulin, fat-soluble vitamins, glucosamine plasma levels, and body composition assessments by DXA (dual-energy X-ray absorptiometry) were secondary endpoints.

## 2. Materials and Methods

### 2.1. Standard Protocol Approvals, Registrations

The clinical study was approved by the Bioethical Committee of the IRCCS San Matteo hospital, Pavia (protocol number: 15012020), and was conducted in accordance with the ethical standards laid down in the 1964 Declaration of Helsinki and its later amendments, with all ICH and ISO international standards (i.e., ICH Harmonized Guideline for GCP E6 (R2); UNI EN ISO 14155:2012 [10]), as well as all the in-force regulations of Regione Lombardia and all national standards of Italy. A written informed consent was obtained from every patient entering the pretreatment phase.

The study was registered with ClinicalTrials.gov (accessed on 21 March 2020) (NCT 04375696), started on 5 May 2020 and concluded on 20 December 2021.

### 2.2. Study Design

The study was a randomized, double-blinded, placebo-controlled, parallel-group study with 90 days of clinical intervention. The two groups were randomly assigned to patients consecutively in chronological order of enrollment, according to the numbering from 1 to 150. The relative product packages provided by the production workshop were identified only with a number from 1 to 150, corresponding to the patient; the composition was only known to the manufacturing workshop.

### 2.3. Participants

The subjects of the study were patients who belonged to the dietetic and endocrinology unit of the Santa Margherita Institute, Azienda di Servizi alla Persona, Pavia, Italy.

### 2.4. Admission and Exclusion Criteria

#### 2.4.1. Admission Criteria

Written informed consent; aged between 18 and 65; both sexes; body mass index (BMI) between 25 and 32 kg/m^2^ and with a weight > 75 kg; absence of previous diet therapy attempts, with at least 5% weight loss in the last year; no fluctuation of at least 3 kg in the previous 3 months; a Beck depression inventory (BDI) score < 20 (probable absence of moderate to severe depressive symptoms); a Binge Eating Scale (BES) score < 27 (probable absence of binge eating disorder).

#### 2.4.2. Exclusion Criteria

Allergy to shellfish or any ingredient in the product; women with presumed or confirmed pregnancy, or in feeding time; subjects with clinically significant cardiopathies, nephropathies, liver diseases, bronchopneumopathies, hemopathies, dermopathies, or chronic degenerative diseases of the central nervous system; subjects with active peptic ulcers, ulcerative colitis, Crohn’s disease, celiac disease, or inflammatory bowel disease; subjects with symptomatic cholelithiasis (at least one episode of biliary colic in the last 6 months) or autoimmune diseases (except autoimmune thyroiditis); subjects with previous or current neoplasms (except skin basalioma diagnosed more than five years previously); subjects with epilepsy (current or previous); obesity secondary to endocrinopathies, or obesity associated with genetic syndromes or other endocrinopathies (except subclinical hypothyroidism with normal TSH values); subjects with significant motor disability or mental retardation; subjects with confirmed or suspected diagnosis of major depressive disorder, bulimia, panic disorder, obsessive–compulsive disorder, post-traumatic stress disorder, bipolar disorder (I or II), or schizophrenia; a previous history or current diagnosis of drug abuse or alcoholism; subjects with changes in smoking habits or that have quit smoking in the last 6 months; subjects that currently use psychoactive drugs, or that have used psychoactive drugs in the last 3 months, or that currently use drugs for chronic diseases affecting body weight or appetite (including: antiobesity drugs), or that have used these drugs in the last 12 months; previous diet therapy attempts, with at least 5% weight loss in the last year and a fluctuation of at least 3 kg in the previous 3 months; subjects with a risk of a cardiovascular event equal to or greater than 20% during the previous 10 years, according to the tables of the Istituto Superiore di Sanità—Heart project; subjects taking medication for dyslipidemia; subjects with TSH levels below or exceeding the normal range, an AST or ALT exceeding 2.5 times the upper limit of normal, or hemoglobin < 11 g/dL, neutrophils > 1500/mm^3^, platelets < 100,000/mm^3^, creatinine > 1.5 mg/dL, or uricemia > 11 mg/dL (therapy with uricosurics or xanthine oxidase inhibitors is allowed, provided that the therapy has already been underway for at least 3 months).

### 2.5. Nutritional Assessment and Nutritional Interventions

A dietician specifically assessed any weight loss compared to the usual weight in good health and in the 6 months prior to the baseline visit.

Both groups were provided with individual counseling and written indications for the execution of a diet therapy in 3 balanced meals equal to 100% of the energy expenditure at baseline (EEB), evaluated by the Harris–Benedict equation, multiplied by the estimated activity factor, with 30% energy from lipids, 60% energy from carbohydrates, and 15% energy from proteins (with a minimum of 0.8 g of protein for the ideal correct weight), with a controlled sodium content and based on the Mediterranean food model.

### 2.6. Anthropometric Measurements

Nutritional status was assessed using anthropometric measurements at the start of the study at baseline (T0), after 45 days (T1), and after 90 days (T2). Body weight and height were measured following a standardized technique [11], and the BMI was calculated (kg/m^2^). The abdominal circumference was also evaluated. Anthropometric parameters were always collected by the same investigator.

### 2.7. Body Composition Assessment

Body composition (fat-free mass (FFM), fat mass (FM)) was measured by dual-energy X-ray absorptiometry (DXA) with the use of a Lunar Prodigy DXA (GE Medical Systems, Chicago, IL, USA). The in vivo coefficients of variation (CVs) were 0.89% and 0.48% for whole-body fat (FM) and FFM, respectively. Visceral adipose tissue volume was estimated using a constant correction factor (0.94 g/cm^3^). The software automatically placed a quadrilateral box, which represented the android region, outlined by the iliac crest, and with a superior height equivalent to 20% of the distance from the top of the iliac crest to the base of the skull [12]. These measurements were applied at baseline (T0) and after 90 days (T2).

### 2.8. Evaluation of Blood Pressure

Blood pressure was assessed at T0 and T2.

### 2.9. Biochemical Variables

The biochemical parameters were assessed at T0 and T2. Venous blood samples were drawn after an overnight fast. Blood samples were obtained through an indwelling catheter inserted in an antecubital vein, and were immediately centrifuged and stored at −80 °C until assayed.

Blood count was measured using a Coulter automated cell counter MAX-M (Beckman Coulter, Inc., Fullerton, CA, USA). Fasting blood glucose (FBG), total cholesterol (TC), low-density lipoprotein cholesterol (LDL-C), high-density lipoprotein cholesterol (HDL-C), and triglyceride (TG) levels were measured by an automatic biochemical analyzer (Hitachi 747, Tokyo, Japan). The serum insulin was evaluated by a double-antibody RIA (Kabi Pharmacia Diagnostics AB, Uppsala, Sweden) and expressed as mcU/mL. The intra- and interassay coefficients of variation were below 6%, and the low-detection limit was 10.7 pmol/L.

To determine insulin resistance, subjects were instructed to fast for 12 h before obtaining the blood samples. Furthermore, the subjects refrained from any form of physical exercise for 48 h before the blood sampling. Insulin resistance was evaluated using the homeostasis model assessment (HOMA) [13]. Finally, for the assessment of safety, routine blood biochemistry parameters of liver and renal function were evaluated: creatinine, alanine aminotransferase, aspartate aminotransferase, and gamma glutamyl transferase were measured with enzymatic–colorimetric methods.

Glucosamine levels were measured using a photometric assay (K-GAMINE 04/18, NEOGEN Europe Ltd., Auchincruive, UK) following the manufacturer’s instructions. Liposoluble vitamin concentrations were determined by reverse-phase high-performance liquid chromatography [14].

### 2.10. Adverse Events

Adverse events were based on spontaneous reporting by subjects, as well as open-ended inquiries by members of the research staff.

### 2.11. Primary and Secondary Endpoints

The primary endpoint was to evaluate the effect of the intake of PG on weight loss in a group of subjects suffering from overweight and mild obesity (BMI between 25 and 32), and with a weight > 75 kg.

The secondary endpoints were to evaluate the effect of PG supplementation on blood levels of triglycerides, TC, HDL-C, LDL-C, glucose, insulin, insulin resistance, liver enzymes (transaminases), creatinine, waist circumference, body composition, serum levels of reactive oxygen species, and the serum antioxidant capacity. Blood levels of fat-soluble vitamins A, D_3_, E, and K_1_, as well as the blood levels of glucosamine, were also assessed.

### 2.12. Treatments

PG and PL were administered at a dosage of 1.5 g/twice a day (total 3 g/day) before the main meals for a period of 3 months. Verum tablets contained 73% polyglucosamine L112, ascorbic and tartaric acid, as well as further tableting aids (PG). PL tablets contained mainly calcium hydrogen phosphate and cellulose, and were colored to mimic the appearance of PG tablets.

### 2.13. Concomitant Medications/Treatments

Concomitant treatments were systematically recorded.

### 2.14. Product Packaging

PG and PL product packages, each consisting of a 3-boxes kit, were identified with a number from 1 to 150 according to the randomization list. The composition of the investigational device was known only to the contract manufacturer so to maintain the complete blinding of the clinical investigation.

### 2.15. Statistical Analysis

The sample size for the ANCOVA was according to Cohen’s approach [15].

Cohen suggested that d = 0.2 is to be considered a ‘small’ effect size, with 0.5 representing a ‘medium’ effect size and 0.8 representing a ‘large’ effect size. This means that, if the difference between two groups’ means is less than 0.2 standard deviations, the difference is negligible, even if it is statistically significant.

For the calculation of the sample sizes, we chose:

α = 0.05; β = 0.20; d = 0.25; St Dev ANCOVA > 3.

These values suggest, for 1 – β ≈ 0.80, the use of N = 150 total patients.

Subgroup analyses were conducted on subjects with a total cholesterol (TC) reduction of >10% and a body weight reduction of ≥5% using the exact chi-square analysis (Fisher test) to determine the differences between groups.

The results will be presented as ITT (intention-to-treat) and PP (per-protocol) values.

For the final evaluation, only the PP values will be considered.

Primary endpoint analysis: the change in weight at the end of the clinical investigation was carried out by the analysis of covariance, adjusting the means of the final values by basal means. The mean difference was reported, with a corresponding SE.

For the secondary endpoints on a continuous scale, the comparison between the intervention groups were approached by the analysis of covariance, adjusting the means of the final values by basal means. The mean difference was reported, with a corresponding standard error. For the baseline values of the comparison in the PP and ITT groups, the ANOVA and Wilcoxon rank sum tests were used.

For the secondary endpoints on the frequencies (the proportion of subjects who improved the intensity of the care profile), the chi-square test and Fisher’s exact test were used. The same analysis was applied for the PP and ITT groups. The missing values were estimated for all subjects undergoing the treatment. The random sampling procedure was adopted: the missing value was replaced by a value extracted randomly from those available for the respective group.

The SAS Institute’s JMP14 Pro 2019 software was used for the analysis.

### 2.16. Compliance

The compliance was determined by counting the residual tablets at the end of the treatment period. Only subjects with at least 95% of the treatment were included in the final evaluation.

The compliance for the diet was controlled with a colloquium between subjects and investigators to adjust the food components in case of discomfort.

### 2.17. Data Quality Assurance

This trial was performed according to the clinical investigation plan, monitored to ensure data quality, and in accordance with the principles of GCP.

## 3. Results

**Figure 1:**
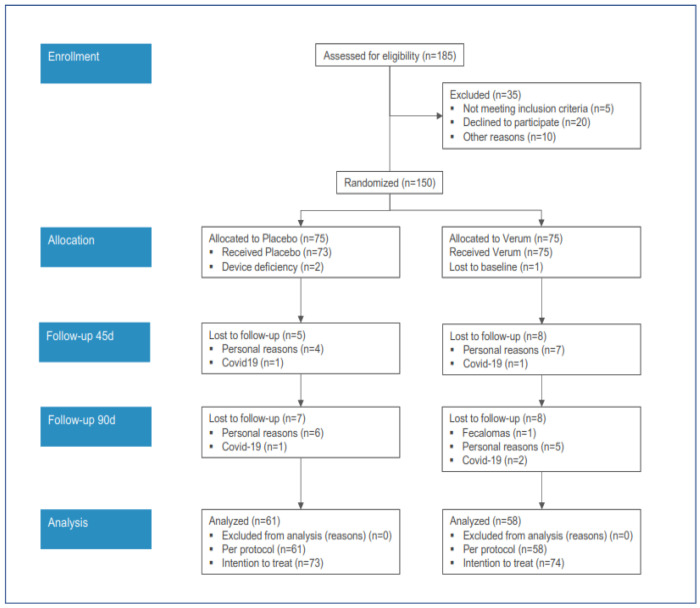
Acquisition of subjects in the clinical study (verum vs. placebo).

The acquisition of subjects for the clinical study is reported in Figure 1.

The PP analysis was conducted on 61 and 58 subjects, respectively, for the PL and PG groups.

Most of the dropouts were due to personal reasons. In two cases administered with the PL, and in three cases administered with the PG, the treatment was withdrawn due to COVID-19. In one case only in the PG group, the treatment was withdrawn due to the onset of fecal impaction (fecalomas).

The compliance was >95% in all subjects.

The clinical results were reported considering the baseline and 90-day values of treatment for the PP (per protocol) subjects only.

However, no differences were found between the PP and ITT (intention-to-treat) measures.

### 3.1. Baseline Values

The general characteristics are summarized in Table 1, while the anthropometric measures are reported in Table 2 for PL and PG.

### 3.2. Clinical Activity

The anthropometric variables significantly modified in the PG group were the BW and BMI. The main variable, BW, showed that the verum administration was significantly more effective than the placebo. Within 3 months, the reduction was 3.71 kg in the PG group and 1.12 kg in the PL group, respectively. Therefore, BW reduction was three times higher in the PG group than in the PL group. VAT reduction was more consistent with PG, although not significant due to the high variability of the baseline data.

Concerning the secondary parameters on the efficacy, the insulin blood levels and HOMA were significantly reduced after 3 months of treatment. In the PG group, insulin blood levels decreased from 14.62 ± 1.05 mcU/mL at baseline to 11.43 ± 0.98 mcU/mL, whereas in the PL group, there was a slight increase from 13.15 ± 1.12 mcU/mL to 13.64 ± 0.66 mcU/mL (*p* = 0.0213 for the covariance analysis). The HOMA ratio changed from 3.59 ± 0.30 to 2.74 ± 0.98 in the PG group and from 3.12 ± 0.30 to 3.27 ± 0.19 in the PL group (*p* = 0.0491 for the covariance analysis). The TC and LDL were not expected to be significantly reduced since the baseline levels were not very high.

Lipid and glucose analysis in blood are reported in Table 3.

None of the vitamins were significantly reduced. However, in both groups, the average levels of vitamin D_3_ and vitamin E were reduced, although still within the normal range.

Glucosamine was not detectable (sensitivity of the method with a detection limit of 0.1 µg/mL). None of the other variables were significantly modified in the two groups.

Concerning safety, blood laboratory analysis was performed and variables related to the organ functionality were collected. There was no significant change observed in the RBC, HB, HCT, PLT, and % Lym. The WBC, especially lymphocytes, increased significantly in the PL group, from 6.54 ± 0.21 × 10^3^ to 6.68 ± 0.12 × 10^3^ and from 2.11 ± 0.07 × 10^3^ to 2.25 ± 0.05 × 10^3^, respectively. In the PG group, no such increase was observed (WBC of 6.33 ± 0.18 × 10^3^ to 6.33 ± 0.12 × 10^3^; Lym of 2.09 ± 0.08 × 10^3^ to 2.07 ± 0.05 × 10^3^). Variables related to organ functionality were AST, ALT, GGT, and creatinine, and were all in the normal range.

### 3.3. Subgroup Analyses

The number of subjects with a clinically relevant decrease >10% of total cholesterol and LDL reduction was compared. The difference was statistically significant in favor of the PG group (with a chi-square of 0.0302); respectively, 17 for PG and 8 for the PL. In addition, the number of subjects showing a BW reduction of ≥5% was significantly higher for the PG group (with a chi-square of 0.0370); respectively, 14 subjects for PG and 6 subjects for the PL.

## 4. Discussion

The present study demonstrates the efficacy of a three-month-long administration with a medical device based on polyglucosamine L112 polymers on the anthropometric and metabolic profile in overweight and obese subjects. In particular, a statistically significant improvement in the primary endpoint was observed in the treated group; in fact, the BW, and consequently the BMI, were significantly reduced. Even the reduction of VAT was more consistent with PG, although the change was not statistically significant.

The results of our study are in agreement with previous studies which have demonstrated that PG was effective for weight loss in combination with dietary interventions [16,17,18]. Even a recent meta-analysis, including 399 overweight and obese subjects, revealed that PG supplementation in conjunction with lifestyle behavioral therapies improves weight loss, BMI, and waist-circumference reduction [9].

Fiber intake is associated with BW reduction [19], and particularly, insoluble fibers with gel-forming capabilities are the most effective since they increase the insulin sensitivity and the transit time of the intestinal bulk [20]. Under these aspects, PG is a particular fiber characterized by a very high fat-binding capacity due to the cationic nature of the polymers and the generation of a gel network which does not allow fat absorption, shifting them down to the colon. The lipases and colonic bacteria release fatty acids and metabolize them into products used for bacterial-membrane formation and as source of energy [21]. Because of this activity, the PG treatment does not cause any steatorrhea and the fat excretion with feces is minimal. Accordingly, in our study, the compliance of treatments was >95% for all subjects. The compliance for the diet was also very satisfactory. In addition, the tolerability of both the PG and PL treatments was similar, with no side effects in the PL group (0%) and one case of fecalomas in the PG group (<2%). In relation to the laboratory variables, the modifications of some of them (WBC and lymphocytes) after the treatments were very limited for both groups and within the normal values. The same happened for the AST and ALT.

In different terms, PG can be considered as a matrix for the slow release of fats, reducing the fat absorption in the upper part of the gut [22] without being absorbed, as confirmed by our finding that the glucosamine levels in the PG group were always below the sensitivity limit of the method of analysis. There are no other medical devices with a similar activity that are a part of bariatric surgery. However, bariatric surgery is not suitable for everyone, and it is not free from complications and side effects [23].

The differences between the ITT and PP analysis were almost identical for both treatments. The main variable of BW reduction in the PG group was three times higher in the PL group (Table 2); the reduction of the baseline values was −3.71 kg and −1.12 kg (covariance analysis, *p* = 0.0352), respectively. In particular, subjects with a ≥5% reduction were significantly higher in the PG group. This is a clinically relevant achievement for the relative short period of treatment. Such results strengthen the outcomes of a previous long-term clinical trial with the same main ingredient (52 weeks). With a similar protocol, investigating PG L112, the BW reduction with verum subjects was 12 kg [24], which is lower, or at the worse comparable, to the 3.7 kg reduction obtained in the present three-month period of administration. However, the changes in the total abdominal fat (TAF), as well as lean and fat mass, were similar in the two groups, probably because a longer period of treatment should be necessary. Nevertheless, the ratio of the risk–benefit of PG, in terms of body-weight reduction (primary objective), was in favor of benefit, since 59% of the subjects showed a reduction of BW of at least 2 kg compared to the PL group, where the same reduction was obtained in only 39% of the subjects. Moreover, considering the <2% of unwanted reactions in the PG group, in this case, the risk–benefit ratio was also in favor of the treatment.

Furthermore, the results of the present study show the efficacy of the PG on insulin blood levels and HOMA, which were significantly reduced after 3 months of treatment in the PG group. This can indicate that the fat-binding capacity of PG can improve insulin sensitivity. This effect is known to be related to the reduction of lipid availability in the diet [22] due to PG’s fat-binding activity in the gut. The insulin reduction is known to be independent from the BW modifications and may indicate that PG is consistent with the prevention of diseases bound to insulin resistance [25].

In relation to total cholesterol levels, the number of cases showing a clinically relevant 10% reduction was significantly higher in the PG group than in the PL group. A previous study was conducted with a similar product, showing that the duration of the treatment is the more important variable to determine the effect on both BW and CH [22].

Finally, a reduction in fat absorption could affect the bioavailability of fat-soluble vitamins. However, previous studies have shown that chitosan does not reduce the absorption of fat-soluble vitamins in obese subjects [26]. The results of our study indicate that PG also does not affect the serum levels of vitamins A, D, E, and K_1_ (Table 4). The reduction of vitamin K_1_ shown in both groups may be ascribable to the diet, which can limit the intake of such vitamins.

Further studies are needed to examine the effects of PG on metabolic parameters and, moreover, studies with a larger sample of subjects need to be conducted. Finally, considering that PG is not absorbed, it would be interesting to evaluate PG use in the pediatric population.

## 5. Conclusions

PG acts as a safe medical device; it is not absorbed and binds lipids in the upper gastrointestinal tract, thus reducing their availability. This effect allows a significant reduction of BW, insulin resistance, and cholesterol levels, without the modification of fat-soluble vitamins.

## Figures and Tables

**Table 1:** General characteristics of the PP (per-protocol) subjects administered with PL and PG; mean ± SE or frequency; (number of cases).

Variable	Treatment (Number of Cases)		ANOVA/X^2^
	PL (61)	PG (58)	*p*-Value
Male	16	15	ns
Female	44	43	ns
BES ^a^	9.7 ± 1.04	10.53 ± 1.01	ns
BDI ^b^	8.5 ± 0.89	9.5 ± 0.89	ns
Age years	50.6 ± 10.76	52.47 ± 1.30	ns
Education 1/2 ^c^	5/56	8/50	ns
Physical activity 0/1/2 ^d^	30/18/13	29/19/10	ns
Smoking 0/1/2 ^e^	50/4/7	49/5/4	ns

Legend: ns = not significant; ^a^ = Binge Eating Scale; ^b^ = Beck depression inventory; ^c^ = 1, secondary school; 2, university degree; ^d^ = 0, sedentary; 1, normal physical activity; 2, active physical activity; ^e^ = 0, never smoked; 1, no smoking for less than 5 years; 2, still smoking.

**Table 2:** Evaluation of the anthropometric variables of subjects administered with PL and PG; mean values ± SE of PP subjects; (number of cases).

Variable	Measure	Treatment (Number of Cases)				Covariance Analysis
		PL (61)		PG (58)		
		Baseline	90 Days	Baseline	90 Days	*p*-Value
H	m	1.67 ± 0.01		1.68 ± 0.01		
BW	kg	83.6 ± 1.28	82.5 ± 0.39	85.0 ± 1.67	81.3 ± 0.40	**0.0352**
BMI	kg/m^2^	30.0 ± 0.25	29.4 ± 0.14	30.1 ± 0.27	28.9 ± 0.14	**0.0265**
AC	cm	102.9 ± 1.22	100.1 ± 0.51	103.4 ± 1.55	100.1 ± 0.52	0.9845
VAT	g	1288 ± 99.7	1216 ± 33.8	1308 ± 102.4	1159 ± 34.7	0.2423
FM	g	35,129 ± 958.5	34,414 ± 479.5	35,784 ± 1251.4	33,486 ± 491.7	0.9409
LM	g	45,993 ± 1007.1	44,999 ± 538.4	46,246 ± 941.7	45,347 ± 556.9	0.6538

Legend: H = height; BW = body weight; BMI = body mass index; AC = abdominal circumference; VAT = visceral abdominal tissue; FM = fat mass; LM = lean mass. Note: Statistically significant data are highlighted in bold type.

**Table 3:** Evaluation of the lipidic and glucose profile of subjects administered with PL and PG; mean values ± SE of PP subjects; (number of cases).

Variable	Measure	Treatment (Number of Cases)				Covariance Analysis
		PL (61)		PG (58)		
		Baseline	90 Days	Baseline	90 Days	*p*-Value
TC	mg/dL	206.13 ± 5.05	205.10 ± 3.00	201.75 ± 4.82	198.52 ± 3.07	0.1294
VLDL	mg/dL	22.70 ± 1.44	22.73 ± 1.25	22.48 ± 1.14	23.04 ± 1.28	0.8612
LDL	mg/dL	126.39 ± 3.90	127.00 ± 2.56	123.17 ± 4.21	121.10 ± 2.62	0.1101
HDL	mg/dL	53.91 ± 1.69	53.39 ± 0.92	52.94 ± 1.71	52.97 ± 0.95	0.7492
TG	mg/dL	113.49 ± 7.19	113.64 ± 6.25	112.38 ± 5.70	115.21 ± 6.41	0.8612
Glucose	mg/dL	92.70 ± 1.32	93.95 ± 1.10	96.71 ± 2.01	94.62 ± 1.13	0.6711
Ins	mcU/mL	13.15 ± 1.12	13.64 ± 0.66	14.62 ± 1.05	11.43 ± 0.98	**0.0213**
HOMA	ratio	3.12 ± 0.30	3.27 ± 0.19	3.59 ± 0.30	2.74 ± 0.19	**0.0491**

Legend: TC = total cholesterol, VLDL = very-low-density lipoproteins; LDL = low-density lipoproteins; HDL = high-density lipoproteins; TG = triglycerides; Ins = insulin; HOMA = homeostasis model assessment Note: Statistically significant data are highlighted in bold type.

**Table 4:** Evaluation of vitamin blood levels in subjects administered with PL and PG; mean values ± SE of PP subjects; (number of cases).

Variable	Measure	PL (61)		PG Verum (58)		Covariance Analysis
		Baseline	90 Days	Baseline	90 Days	*p*-Value
Vitamin A	µg/mL	0.36 ± 0.02	0.35 ± 0.01	0.39 ± 0.02	0.34 ± 0.02	0.6897
Vitamin E	µg/mL	7.69 ± 0.27	7.52 ± 0.19	7.37 ± 0.24	7.38 ± 0.20	0.6158
Vitamin D_3_	ng/mL	25.38 ± 1.47	22.19 ± 1.13	25.61 ± 1.66	21.86 ± 1.16	0.8412
Vitamin K_1_	ng/mL	1.15 ± 0.09	0.87 ± 0.06	1.32 ± 0.12	0.79 ± 0.06	0.3239

## Data Availability

The data presented in this study are available on request from the corresponding author.
